# Time-Correction Model Based on Diverter Speed for a pVTt Gas Flow Primary Standard

**DOI:** 10.3390/s22114001

**Published:** 2022-05-25

**Authors:** Primož Žibret, Gregor Bobovnik, Jože Kutin

**Affiliations:** Laboratory of Measurements in Process Engineering, Faculty of Mechanical Engineering, University of Ljubljana, Aškerčeva 6, SI-1000 Ljubljana, Slovenia; gregor.bobovnik@fs.uni-lj.si (G.B.); joze.kutin@fs.uni-lj.si (J.K.)

**Keywords:** flow rate measurement, pVTt, time correction, diverter

## Abstract

The volumetric primary standard with a constant volume (pVTt) determines the flow rate from the rate of change of gas density in the defined measuring volume. The key element of the measuring system that uses the flying start–stop method is a diverter. This paper presents a time correction model that adapts the correction according to the diverting speed implemented in our pVTt system developed for flow rates smaller than $12\;mg\min^{-1}$. A detailed study of the diverter’s effects on the effective collection time and two methods for determining the time correction are presented. One is based on the ISO 4185 standard and the other on measurements of the dynamic pressure upstream of the diverter. A set of correction time measurements were made at different diversion speeds to define the correction model at a flow rate of $9\;mg\min^{-1}$. The results show very good agreement between the measurements with both methods and the defined correction model. Additional measurements were made at smaller flow rates and the results indicate the independence of the time correction from the measured flow rate.

## 1. Introduction

The current metrological capabilities for the gas flow measurements of the Laboratory for Measurement in Process Engineering at the Faculty of Mechanical Engineering, University of Ljubljana cover the range down to $1.2\;mg\min^{-1}$ using the piston prover primary standard as the reference device. The expanded uncertainty of measurement does not exceed 0.15% for flow rates above $12\;mg\min^{-1}$, but it increases to 1% at smaller flow rates down to $1.2\;mg\min^{-1}$ [1]. With the intention to expand the measuring range and to decrease measurement uncertainties at the lowest flow rates, the new constant volume primary standard has been developed, covering the range between $0.12\;mg\min^{-1}$ and $12\;mg\min^{-1}$ with the target expanded uncertainty of 0.2%. The development goals has also been to keep the time interval of measurements below a few minutes and to minimize the possibility of human errors by fully automating the measuring system.

Primary standards for flow measurements all rest on the fundamental physics of mass accumulation in a certain time window. When the fluid is a gas, there are two basic groups of primary standards. Gravimetric standards use scales or mass comparators to evaluate the mass change of a depleted/filled pressure vessel [2,3,4,5] with the best uncertainties defined as 0.019% [2] of the measured flow rate. The volumetric method uses the definition of gas mass as a product of the gas density and a known volume that the gas occupies. The latter methods are further divided into methods with changing volume [5,6,7,8,9,10,11,12,13,14,15] and methods with constant volume (changed density), usually also labelled with the acronym pVTt (pressure, volume, temperature, time) [16,17,18,19,20,21,22,23,24] with uncertainties going down to 0.013% [6] and 0.015% [22] of measured flow rate respectively. In the former the volume can be changed with the translation of a sealed piston inside a known cylinder or the translation of a liquid-sealed bell with a known diameter. In both cases the time needed for a piston/bell to travel between the two points on the path is recorded to obtain the change of volume with time. That, combined with the measured density, theoretically kept constant, gives an indication of the mass flow. When using a constant volume, the result of the usually entering gas is predominantly expressed through a pressure change, from which the density change is evaluated using the gas equation. Again, using the measured density change in the recorded time window with a fixed and known volume we can determine the mass flow of the gas.

We focused on a volumetric method with a constant volume (pVTt) and a static method of mass determination that uses a flying start–stop that guarantees the time for the flow stabilization. One of the key parts of this method is the use of a diverter, which diverts the flow in/out of the measuring volume $V_{\mathrm{mea}}$ and is also used as an interrupter for the time measurement [25]. The basic measurement model is as follows:

(1) $$q_{m}=\frac{V_{\mathrm{mea}}}{t_{\mathrm{ef}}}\Delta \rho =\frac{V_{\mathrm{mea}}}{t_{\mathrm{ef}}}\left(\rho_{\mathrm{end}}\left(p_{\mathrm{end}},T_{\mathrm{end}}\right)-\rho_{\mathrm{st}}\left(p_{\mathrm{st}},T_{\mathrm{st}}\right)\right).$$

The densities of the confined gas at the start $\rho_{\mathrm{st}}$ and the end $\rho_{\mathrm{end}}$ correspond to the measured pressure *p* and the temperature *T* at the start and the end of the measurement in stable conditions. The system is collecting mass, and the time window, when the flow is directed into the system, is called the effective collection time $t_{\mathrm{ef}}$. It is defined in a way that the equality between the time integral of the actual flow into the $V_{\mathrm{mea}}$ and the product of the determined average flow rate with $t_{\mathrm{ef}}$ is assured. This is determined as the sum of the measured collection time $t_{\mathrm{mea}}$ and the time correction $t_{\mathrm{cor}}$ that compensates for features of the actual system,

(2) $$t_{\mathrm{ef}}=t_{\mathrm{mea}}+t_{\mathrm{cor}}.$$

The aim of this article is to evaluate two methods for determining the time correction and set up a new time correction model that has the possibility to take into account potential changes in the operating speed of the diverter. The first method is derived from the ISO 4185 [26] standard and based on measuring the same flow rate at different collection times (termed Method 1). Besides, we propose a novel method of determining the time correction based on the measured dynamic pressure signals upstream of the diverter (termed Method 2). The target measurement uncertainty of the correction time is 0.01s or less.

In the Section 2, we will first describe the theoretical operation of the diverter and its influence on the correction time. The following is a description of the measuring system in Section 3. In Section 4, the methods for measuring the correction time will be defined, and the effects on the result will be presented. In Section 5, a correction model according to Method 1 will be obtained on the basis of measurements. It will be followed by Section 6, where Method 2 will be validated and used to check the dependence of the correction time on the flow rate. The article ends with Section 7 that summarises the main contributions.

## 2. Physical Background of the Flow Diversion

**Figure 1:**
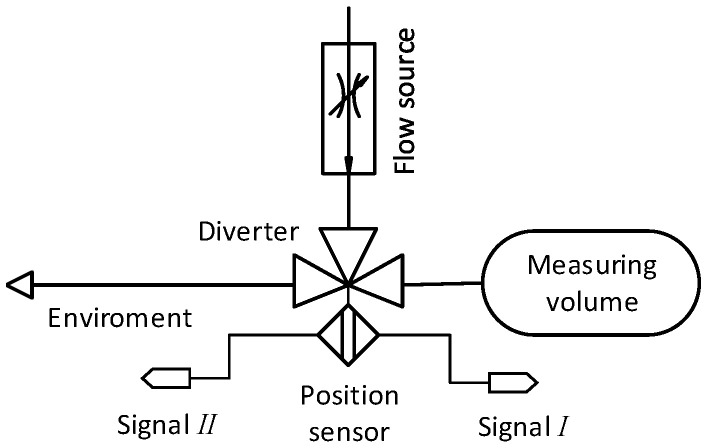
Schematic representation of a diverter.

The diverter used for the flying start–stop method for the flow measurement is a mechanical device that diverts the flow from one inlet between two outlets. This is achieved by moving a diverting element between two end positions. The diverter shown in Figure 1 is equipped with a proximity sensor that generates Signal *I* and Signal II, high levels of which represent the flow path opened to the measuring volume and to the environment, respectively. The position signals are used as a source for the time measurement.

**Figure 2:**
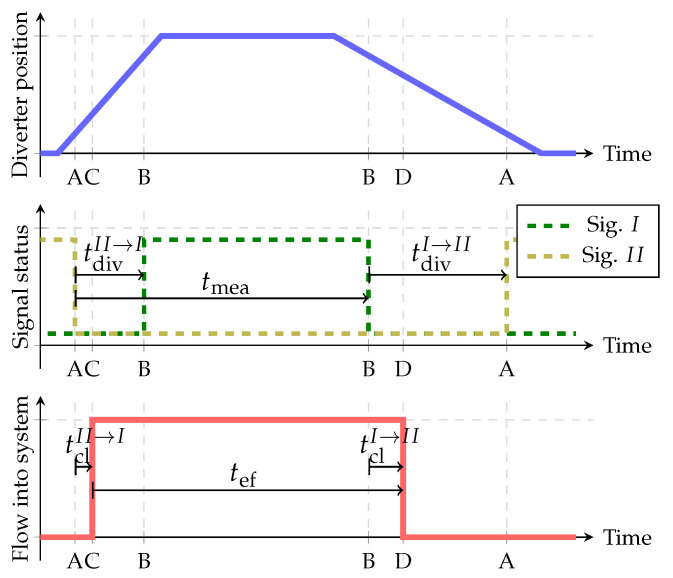
Connection between diverter position, position sensor signals and mass flow into measuring volume.

While the diverter moves from one end position to another, the position signals change accordingly (Figure 2). For most of the time during the diverter’s motion both signals are at a low level. We identified the diversion time $t_{\mathrm{div}}$ as the time between the drop of one signal to a low level and the rise of the other signal to a high level. In Figure 2, changes of Signal *I* are labelled with A and changes of Signal II with B.

During the flow measurement, the diverter always changes its state (position) twice; it returns to the initial position. At the beginning of the measurement the gas accumulation begins when the diverter outlet to the environment is closed (labelled with timestamp C in Figure 2). The mass accumulation finishes when the diverter outlet to the measuring volume closes (labelled with time stamp D in Figure 2). As demonstrated in Figure 2, the measured time $t_{\mathrm{mea}}$ is defined as the time between two consecutive drops of Signal II (first timestamp A in Figure 2) and Signal *I* (second timestamp B in Figure 2). The time delay between the signal drop and the actual diversion (between timestamps A and C at the beginning and between B and D at the end) can be defined as the closing time $t_{\mathrm{cl}}$. So $t_{\mathrm{ef}}$ can be expressed as:

(3) $$t_{\mathrm{ef}}=t_{\mathrm{mea}}-t_{\mathrm{cl}}^{II\to I}+t_{\mathrm{cl}}^{I\to II}.$$

If we consider Equation (2) the correction time equals:

(4) $$t_{\mathrm{cor}}=t_{\mathrm{cl}}^{I\to II}-t_{\mathrm{cl}}^{II\to I}.$$

Under real-life conditions, the diverter’s actions in the opposite directions are never fully symmetrical. The correction time is a direct consequence of this asymmetrical error.

## 3. Measurement System

**Figure 3:**
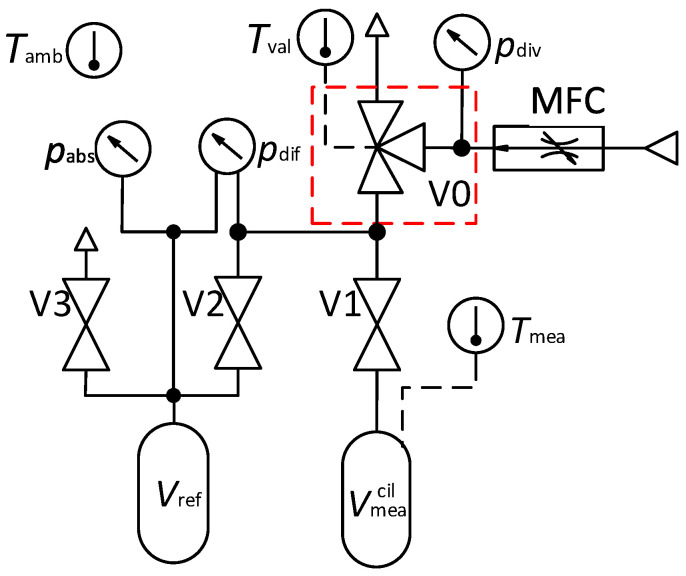
Schematic of a system without control signals. Diverter (V0) in red box.

**Figure 4:**
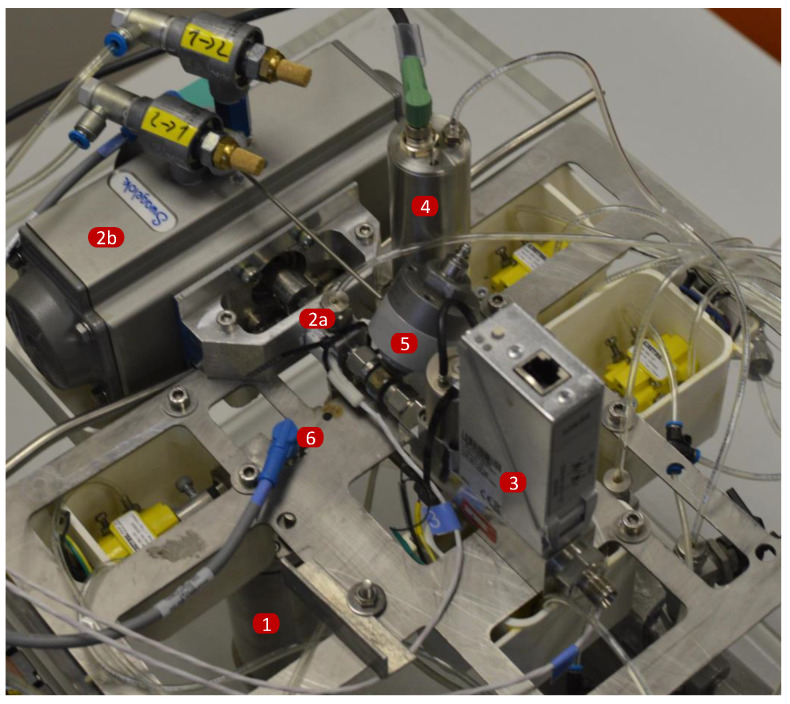
Photograph of the system taken during maintenance. Labelled essential components: (1)—Cylinder part of $V_{\mathrm{mea}}$, (2a)—valve V0, (2b)—actuator for V0, (3)—mass flow contoller, (4)—$p_{\mathrm{dif}}$ pressure transducer, (5)—$p_{\mathrm{div}}$ pressure sensor, (6)—$T_{\mathrm{mea}}$ temperature probe.

A basic schematic of the measurement system is presented in Figure 3, accompanied by a photograph of the system (Figure 4). All the measurements reported in this paper were performed with dry air as the measured gas. The mass flow controller (Bronkhorst, F-201CV-020) is used as a stable flow source. The system operates at atmospheric pressure with relatively small pressure changes of up to a few kPa downstream of the mass flow controller, which minimises the effect on its stability. The diverter is a three-way ball valve (V0) with a pneumatic actuation (Swagelok, SS-41GXS3MM-A15XD). The actuator’s speed was controlled with a pressure regulator to define the driving pressure and two variable flow restrictions installed on each of the actuator’s driving ports. The diverter is equipped with an inductive sensor (Pepperl + Fuchs, NBN3-F25-E8-V1-3D) that generates two output signals, each for one end position of the actuator. The signals are acquired using a DAQ card (NI, USB-6341) with an on-board timer for the time measurement. For enhanced monitoring of the diverter’s operation, an additional piezo pressure sensor $p_{\mathrm{div}}$ (Kistler, 7261 & 5007) is added upstream of the diverter.

The measuring volume $V_{\mathrm{mea}}$ is a sum of the calibrated cylinder $V_{\mathrm{mea}}^{\mathrm{cil}}$ and the connection tubing $V_{\mathrm{mea}}^{\mathrm{tub}}$. The volume of the cylinder ($99.96\;cm^{3}$) is based on a traceable calibration of its dimensions. The volume of the tubing connecting the mass flow controller, the diverter, the pressure sensors and the cylinder ($4.73\;cm^{3}$) was determined using the method of volume expansion [16].

For optimal thermal stability, the calibrated cylinder and most of the tubing are submerged in a water bath. To reduce the unnecessary heat sources, all the valves (V1–V3) in direct contact with the measured gas are pneumatically actuated. The entire system is inserted into the climatic chamber and is maintained at a constant temperature. The temperatures at three locations are monitored during the operation of the system. To monitor the temperature of the cylinder $T_{\mathrm{mea}}$, a cylindrical probe is inserted into a hole in the cylinder’s wall (TetraTec Instruments, WIT-S/Pico Technology, PT-104). The temperature of the diverter $T_{\mathrm{val}}$ is monitored with a surface probe attached to it (Omega, SA2F-RTD/Pico Technology, PT-104). To measure the temperature inside the climatic chamber $T_{\mathrm{amb}}$ a cylindrical probe is exposed to the ambient air (TetraTec Instruments, WIT-S/Pico Technology, PT-104).

The pressure inside $V_{\mathrm{mea}}$ is determined by a combination of differential $p_{\mathrm{dif}}$ (MENSOR CPT9000) and absolute $p_{\mathrm{abs}}$ (MENSOR CPG2500) pressure transducers. The maximum collected mass of air is about 3.1mg and is defined by the size of the measuring volume $V_{\mathrm{mea}}$ and the range of the differential pressure transducer up to 2.5kPa, Differential pressure transducer measures the pressure difference between the measuring volume $V_{\mathrm{mea}}$ and the reference volume $V_{\mathrm{ref}}$. Both volumes are first connected and isolated from the atmosphere using valve V3 and later separated one from another using valve V2 (Figure 3). By immersing the reference volume into the water bath kept at a constant temperature, any changes of the reference pressure $p_{\mathrm{ref}}$ due to the temperature instability are being minimised. The gas pressures in the measuring volume at the start and the end of the measurement cycle are determined in stable conditions by considering appropriate temperature-stabilisation times.

All the measured signals were processed and recorded on a PC in a LabVIEW environment. From the measured temperature and pressure the densities inside $V_{\mathrm{mea}}$ are calculated using the REFPROP database [27]. With LabVIEW the whole measurement process is automated and controlled with the help of a PLC (Controllino MAXI) and a series of solenoid valves, which drive the pneumatic actuators.

## 4. Measurement Methods for the Correction Time

### 4.1. Method 1: Varying the Collection Time

The basic idea of this method follows the method defined in ISO 4185 [26] for measurements of liquid flow, which suggests conducting two measurements, first using a single continuous collection of the mass and second a collection with multiple diverter actions in between. We adjusted it to better adapt to our system based on two assumptions. First, is that two consecutive measurements of a stable flow using different collection times have to indicate the same flow-rate reading. Second, is that the only source of the correction time is the diverting valve, operation of which is repeatable for both measurements. Applying Equation (1) for two conducted measurements, we can express the correction time as:

(5) $$t_{\mathrm{cor}}^{M1}=\frac{\Delta \rho_{t_{1}}t_{\mathrm{mea}\;t_{2}}-\Delta \rho_{t_{2}}t_{\mathrm{mea}\;t_{1}}}{\Delta \rho_{t_{2}}-\Delta \rho_{t_{1}}},$$

where the indexes $t_{1}$ and $t_{2}$ correspond to two consecutive flow measurements of a constant mass flow rate with different collection times.

The measurement system was programmed to continuously measure the flow rate with an alternating collection time. The correction time with Method 1 was calculated after each flow measurement (except for after the first one) from the last two consecutive flow measurements.

### 4.2. Method 2: Closing-Time Determination Using a Pressure Sensor

The chosen diverting valve closes both outlets for a brief period of time (zero crossover). During the diversion, when both outlets of the diverting valve are closed, gas is accumulated in the volume upstream of the diverting valve (V0). After one of the valve outlets opens (into the measuring volume or into the environment) the accumulated gas is released. The effective time determination based on the matching densities upstream of the diverter at the start and end diversion is described in [16]. We decided to use a different approach based on the same effect.

**Figure 5:**
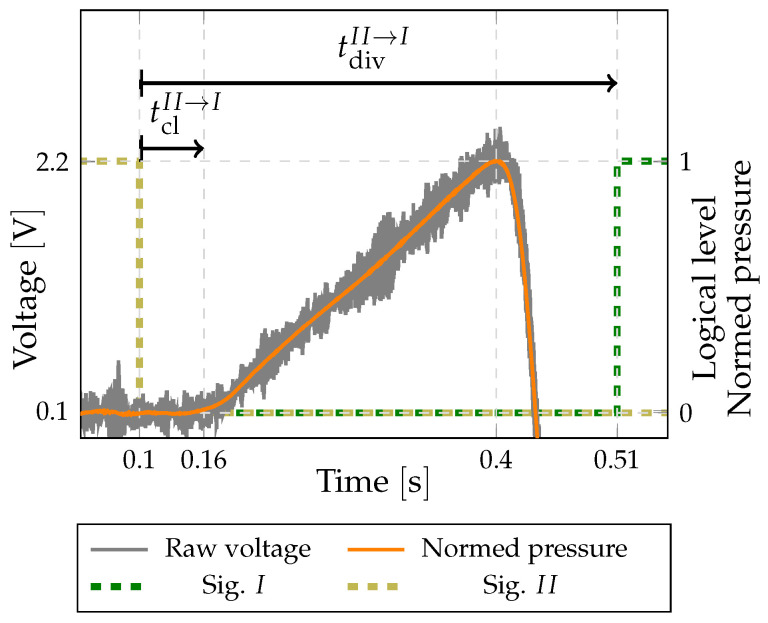
Example of recorded voltage from the piezo pressure sensor together with signals from inductive sensor.

In order to record the accumulation of the gas upstream of the diverter and define the exact moment of the outlet closure we used a piezo pressure sensor. An example of the acquired voltage signal at the start of the collection time is shown in Figure 5. For a further evaluation the raw voltage signal from the sensor is filtered and normalised. A low-pass filter is used to eliminate the electrical noise and the structural vibrations. It plays a crucial part during flows in the lower part of the system-measurement range where the signal-to-noise level becomes worse. Since the data capture in this case is triggered with Signal II (pre-trigger at 0.1s with falling edge) and $t_{\mathrm{div}}^{II\to I}$ measured with the counter (two edge-separation measurements), we can synchronize signals from the proximity and the piezo pressure sensor (Figure 5). As diversion starts, Signal II from the proximity sensor drops to a low value. When the valve closes, the signal from the pressure sensor starts to rise and after an initial transition it changes linearly. When the valve opens on the other outlet, the gas is released and a pressure drop is recorded. To define the moment at which the diverter closes, the gas flow to the initially open outlet, we fit a linear-regression model to the linear part of the rising pressure. The time when the regression line crosses the initial base level of the signal is defined as the moment of the valve’s closure. Consequently, $t_{\mathrm{cl}}^{II\to I}$ can also be defined as shown in Figure 5. The same procedure is made for the end diversion, and the combination of the two consecutive $t_{\mathrm{cl}}$ gives us $t_{\mathrm{cor}}^{M2}$ according to Equation (4). The evaluated moments of closure are probably not exact, but since we are evaluating the asymmetry between two consecutive diversions, this introduces a negligible error when calculating the value of $t_{\mathrm{cor}}^{M2}$ as confirmed by the comparison of both methods in Section 6.

### 4.3. Effects on Measurement of the Correction Time

The experimental results in this section were obtained for a flow rate of $q_{m}\approx 9\;mg\min^{-1}$ and with the diverter set to operate with $t_{\mathrm{div}}^{II\to I}\approx 0.2\;s$ and $t_{\mathrm{div}}^{I\to II}\approx 0.6\;s$. The system was programmed to alternate between collection times of 10s and 20s, which correspond to approximately half and the maximum value of the collected mass of the gas for the given flow rate, respectively.

**Figure 6:**
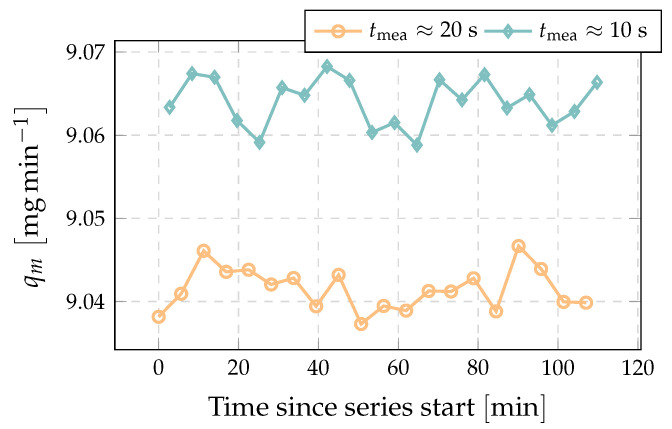
Example of consecutively measured flow rate with alternating fill time and without time correction ($t_{\mathrm{div}}^{II\to I}\approx 0.2\;s$ and $t_{\mathrm{div}}^{I\to II}\approx 0.6\;s$).

In Figure 6 the effect of different measurement times on the measured flow rate without time correction, $t_{\mathrm{ef}}$ is $t_{\mathrm{mea}}$, is evident. The measured flow rate at the shorter collection time is on average higher by 0.25%. This means that the actual flow rate is even lower than the measured value for the longer collection time and so the time correction needs to be added to the measured collection time $t_{\mathrm{mea}}$.

**Figure 7:**
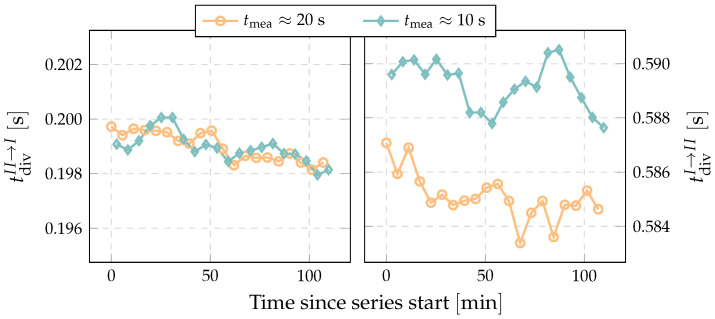
Example of consecutively measured diversion time ($t_{\mathrm{div}}^{II\to I}\approx 0.2\;s$ and $t_{\mathrm{div}}^{I\to II}\approx 0.6\;s$; $q_{m}\approx 9\;mg\min^{-1}$).

Figure 7 shows the stability of the diverter’s operation. It can be seen that even if all the settings remained unchanged, the diverter speed changes slightly (0.002s). At the start of the diversion there is no difference for the different collection times, but at the end diversion there is a difference of about 0.005s. Additional measurements of the diverter’s driving pressure showed that this is related to the ability of the pressure regulator to quickly reach the pressure set point after the diversion and to the stability of the pressure drop on the flow restrictor.

**Figure 8:**
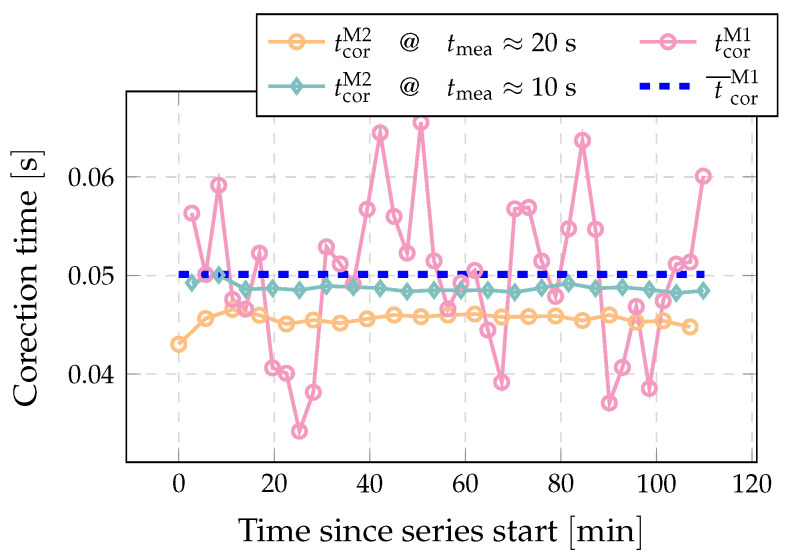
Example of consecutively measured correction time with two methods. Blue lines represent average of presented Method 1 results. ($t_{\mathrm{div}}^{II\to I}\approx 0.2\;s$ and $t_{\mathrm{div}}^{I\to II}\approx 0.6\;s$; $q_{m}\approx 9\;mg\min^{-1}$).

Figure 8 shows the results of the correction time measurements according to both Method 1 and Method 2. It can be seen that Method 2 successfully identifies the change in the correction time between shorter and longer measurements due to the previously mentioned changes in the diverter’s operation. For Method 1, which is based on the assumption of an unchanged diverter operation during both required measurements, this change in diverter operation is a potential source of measurement uncertainty.

The combined standard uncertainty of the correction time according to Method 1 can be estimated as [28]:

(6) $$u\left(t_{\mathrm{cor}}^{M1}\right)=\sqrt{u_{t_{\mathrm{div}}}^{2}\left(t_{\mathrm{cor}}^{M1}\right)+u_{p_{\mathrm{dif}}}^{2}\left(t_{\mathrm{cor}}^{M1}\right)+u_{q_{m}}^{2}\left(t_{\mathrm{cor}}^{M1}\right)+u_{\mathrm{leak}}^{2}\left(t_{\mathrm{cor}}^{M1}\right)+u_{\mathrm{rep}}^{2}\left(t_{\mathrm{cor}}^{M1}\right)},$$

taking into account following uncertainty contributions: the discussed change in the diverter’s operation $u_{t_{\mathrm{div}}}\left(t_{\mathrm{cor}}^{M1}\right)$, the uncertainty of the measured differential pressure $u_{p_{\mathrm{dif}}}\left(t_{\mathrm{cor}}^{M1}\right)$, the stability of the generated flow $u_{q_{m}}\left(t_{\mathrm{cor}}^{M1}\right)$, the leakage $u_{\mathrm{leak}}\left(t_{\mathrm{cor}}^{M1}\right)$ and the repeatability $u_{\mathrm{rep}}\left(t_{\mathrm{cor}}^{M1}\right)$. As a conservative estimate of the change in the correction time due to the diverter’s instability, a quarter of the change in diversion time $t_{\mathrm{div}}^{I\to II}$ between two consecutive measurements was assumed. With the maximum change in the diversion time being 0.008s (Figure 7), the uncertainty introduced by the diverter’s stability comes to $u_{t_{\mathrm{div}}}\left(t_{\mathrm{cor}}^{M1}\right)=0.002\;s$. For the collected masses in the presented measurement the non-linearity of the differential pressure transducer (0.2Pa) introduces an uncertainty of about $u_{f_{\mathrm{dif}}}\left(t_{\mathrm{cor}}^{M1}\right)=0.003\;s$. Assuming a stability of the generated flow rate between two consecutive measurements of about 0.01%, the related uncertainty comes to $u_{q_{m}}\left(t_{\mathrm{cor}}^{M1}\right)=0.001\;s$. The estimated leakage flow rate of less than $1.2\times 10^{-4}\;mg\min^{-1}$ has a negligible impact on Method 1 at this flow rate. The repeatability is estimated by the experimental standard deviation of the mean [28]:

(7) $$u_{\mathrm{rep}}\left(t_{\mathrm{cor}}^{M1}\right)=\frac{s(t_{\mathrm{cor}}^{M1})}{\sqrt{n}},$$

where $s(t_{\mathrm{cor}}^{M1})$ is the experimental standard deviation and *n* number of measurements. For forty consecutive measurements $u_{\mathrm{rep}}\left(t_{\mathrm{cor}}^{M1}\right)$ equals about 0.001s. All these impacts combined give a standard uncertainty of less than 0.004s. Considering the lower gas flows, the uncertainty increases, most significantly due to the higher impact of the non-linearity of the pressure sensor. For the example at $1\;mg\min^{-1}$ the standard uncertainty of the correction time increases to 0.01s and at $0.5\;mg\min^{-1}$ even to 0.02s, which makes Method 1 of limited use in a determination of the correction time at flow rates below $1\;mg\min^{-1}$.

Contrary to Method 1, the uncertainty of the correction time according to Method 2 cannot be explicitly estimated. The results for Method 2 were therefore validated upon comparison with the results of Method 1 (see Section 6).

## 5. Model for the Correction Time’s Dependency on the Diverter’s Speed

The purpose is to define the model for the correction time that considers potential changes in the speed of the diverter. The correction time is expected to depend linearly on the start and end diversion times, so the following linear model is proposed:

(8) $$t_{\mathrm{cor}}^{\mathrm{lm}}=k^{I\to II}\;\cdot \;t_{\mathrm{div}}^{I\to II}-k^{II\to I}\;\cdot \;t_{\mathrm{div}}^{II\to I},$$

with two model coefficients $k^{I\to II}$ and $k^{II\to I}$.

The correction time was measured at a flow rate of $9\;mg\min^{-1}$ and four different diverter setups related to different combinations of the start and the end diversion times. We selected two different speeds of the diverter operation, i.e., fast ($t_{\mathrm{div}}\approx 0.2\;s$) and slow ($t_{\mathrm{div}}\approx \;0.6\;s$), which gave us four combinations to test. For each diverter setup, at least forty consecutive measurements were made. Applying linear regression to the measurement results for the correction time determined by Method 1 and the corresponding diversion times we obtain the model:

(9) $$t_{\mathrm{cor}}^{\mathrm{lm}}=0.157\;\cdot \;t_{\mathrm{div}}^{I\to II}-0.203\;\cdot \;t_{\mathrm{div}}^{II\to I}.$$

The standard uncertainty of the approximation is 0.009s. Together with the standard uncertainty discussed in the previous section, we obtained a standard uncertainty of the correction time determined by the model of 0.010s.

**Figure 9:**
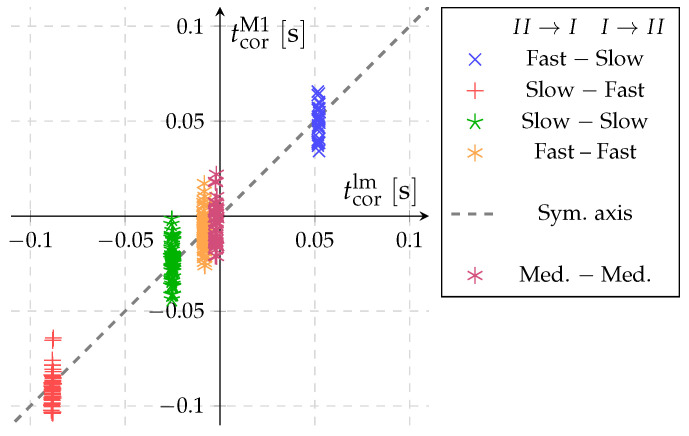
Comparison of the measurement results for the correction time according to Method 1 and the approximation model Equation (9) ($q_{m}\approx 9\;mg\min^{-1}$).

The measured correction times versus their approximate values according to Equation (9) are presented in Figure 9. Figure 9 also shows an additional series of measurements performed at the medium diverter speed (Med. − Med.; $t_{\mathrm{div}}\approx 0.4\;s$), which was used for a linearity check of the obtained model. This fifth combination (Med. − Med.) also shows good agreement with the model and confirms its validity.

## 6. Validation of Pressure Based Method for the Time Correction (Method 2)

**Figure 10:**
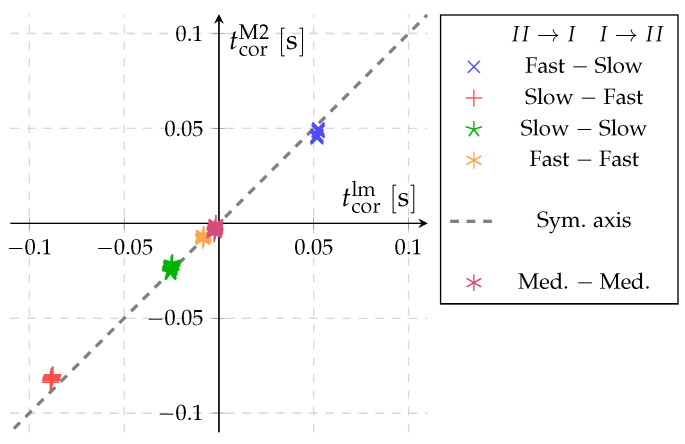
Comparison of the measurement results for the correction time according to Method 2 and the approximation model Equation (9) ($q_{m}\approx 9\;mg\min^{-1}$).

Figure 10 shows a comparison of the correction times obtained with pressure based method (Method 2) versus the approximated values using Equation (9) that is based on Method 1. The same sets of measurements as in Figure 9 are presented. The obtained data show that the values for all five diverter setups are lying close to the symmetry axis. The largest deviation of Method 2 and the model Equation (9) is 0.008s at the Slow–Fast setup of diverter. For values where the diverter travels with approximately the same speed in both directions, the difference does not exceed 0.002s. Since the deviations are smaller than the standard uncertainty of the correction model, we can conclude that Method 2 produces results that are consistent with Method 1, without significant systematic deviations. The application of Method 2 has some important advantages. Comparing Figure 9 and Figure 10, Method 2 shows significantly lower scatter for the determined time corrections compared to Method 1. It is also a faster method, because it requires only two consecutive switches of the diverter instead of performing two full flow-rate measurements using Method 1.

Additional measurements at the lower flow rates of $q_{m}\approx 1.1\;mg\min^{-1}$ and $q_{m}\approx 0.5\;mg\min^{-1}$ were made to investigate the effect of the flow rate on the correction time. Since the uncertainty of Method 1 at lower flow rate rises substantially, we decided to use only Method 2. The Slow–Slow diverter setup was selected. The system was operating with the collection time fixed for each flow rate.

**Figure 11:**
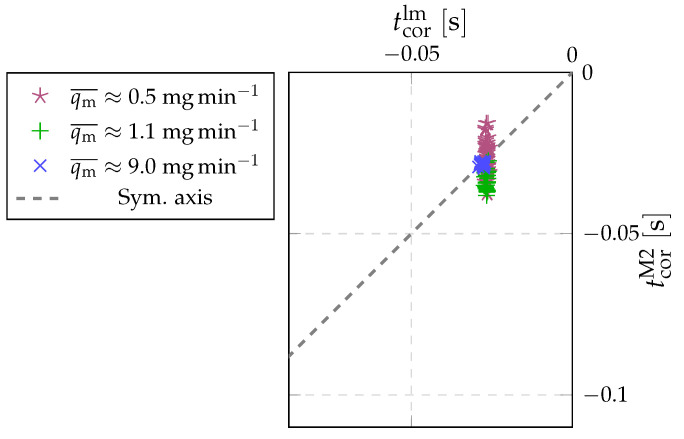
Results of correction time measurement obtained with Method 2 in combination with values according to defined model for different flow rates.

The results of three successive measurement series at different flow rates are presented in Figure 11. From a value of 0.0005s at $9\;mg\min^{-1}$ the standard deviation rises to 0.003s at $1.1\;mg\min^{-1}$ and further to 0.005s at $0.5\;mg\min^{-1}$. The increase of the standard deviation rise comes from the fact that the observed pressure increase is substantially smaller and so the signal-to-noise ratio becomes worse. By comparing Figure 11 to Figure 9 we can observe that even the scatter of Method 2 at the lowest flow rate is still smaller than the scatter of Method 1 at $9\;mg\min^{-1}$.

However, if we look at the average values, we see that at a flow rate of $1.1\;mg\min^{-1}$, there was a 0.007s deviation between the average values of the model and Method 2, whereas at a flow rate of $0.5\;mg\min^{-1}$, the averages coincide within 0.001s. This indicates a systematic shift at $1.1\;mg\min^{-1}$, which is probably not due to the changed flow rate. However, given that the deviation is still within the measurement uncertainty of the model, we can conclude that the time correction is not very dependent on the measured flow rate.

## 7. Conclusions

A primary standard based on a constant-volume volumetric method (pVTt) using the flying start–stop method with a static determination of the captured mass was presented. The flying start–stop method greatly depends on the operation of the diverter, which is the main source of the necessary correction for the measured time. The uncertainty of the time correction is a major contribution to the system uncertainty when using short collection times. For example, 0.05s of time correction uncertainty leads to the uncertainty of 0.5% of the measured flow rate when the collection time equals 10s. We have defined and compared two methods for determining the time correction. Method 1, based on the ISO 4185 standard [26], considers that the measured mass flow should be independent of the change in the collection time. Based on a detailed analysis of the operation of the diverter, we defined a new method of determining the correction time, Method 2, which is based on measuring the pressure change upstream of the diverter linked to the diverter position.

Using Method 1 and controlling the operation of the diverter, the linear measurement model for the correction time was determined, which takes into account changes in the speed of the diverter. The uncertainty analysis of the resulting correction time took into account the uncertainty contributions related to the application of Method 1 (flow instability, diverter instability, non-linearity of the pressure transducer, repeatability) and the uncertainty of the model approximation. It was estimated that the standard uncertainty of the model does not exceed 0.01s. A comparison of the time-correction results obtained using both methods confirms the applicability of Method 2. Method 2 was also used to evaluate the time correction for smaller flow rates, where Method 1 experiences a significant increase in the uncertainty. The results show a minor effect of the flow rate on the time correction.

A potential source of the observed dependency of the diverter’s operation on the time between the two diversions is the stability of the driving pressure of the diverter. For that reason, the plan for the future is to improve the configuration of this part of the measurement system and re-evaluate both methods for the correction time. The uncertainty of the model for the correction time could be further decreased by defining a narrower speed range for the diverter during the use of the PVTt primary standard.

## Data Availability

Not applicable.
